# *“Never at ease” –* family carers within integrated palliative care: a multinational, mixed method study

**DOI:** 10.1186/s12904-018-0291-7

**Published:** 2018-03-01

**Authors:** Gülay Ateş, Anne Frederieke Ebenau, Csilla Busa, Ágnes Csikos, Jeroen Hasselaar, Birgit Jaspers, Johan Menten, Sheila Payne, Karen Van Beek, Sandra Varey, Marieke Groot, Lukas Radbruch

**Affiliations:** 1 Department of Palliative Medicine, University Hospital Bonn, Sigmund-Freud-Straße 25, 53127 Bonn, Germany; 2 Department of Anesthesiology, Pain and Palliative Medicine, Radboud University Nijmegen Medical Center, P.O. Box 9101 (internal code 549), 6500 Nijmegen, HB Netherlands; 3 Radiation Oncology Department and Palliative Care, University Hospital Leuven, Herestraat 49, 3000 Leuven, Belgium; 4 Division of Health Research, International Observatory on End of Life Care, Lancaster University, Furness Building, Lancaster, LA1 4YG UK; 5 Department of Hospice and Palliative Care, Institution of Primary Health Care, University of Pecs Medical School, Szigeti str 12, Pécs, H-7624 Hungary; 6 Center of Palliative Care, Malteser Hospital Seliger Gerhard Bonn/Rhein-Sieg, Von-Hompesch-Straße 1, Bonn, 53123 Germany

**Keywords:** Palliative care, Family carers, Integrated palliative care, Burden, Rewards, Mixed methods, End-of-life, Family caregiving

## Abstract

**Background:**

Family carers manage a wide range of responsibilities in the lives and care of patients receiving palliative care. They fulfil multiple roles and perform activities within different settings. This has immediate consequences on family carers’ every-day lives. According to literature, family carers in palliative care are both part of the formal and informal care network, but also persons in need of support. This article aims to investigate 1) burdens and rewards associated with family caregiving and 2) what family carers find helpful in their contact with professionals from integrated palliative care initiatives (IPC-i) and other services.

**Methods:**

Family carers looking after patients with cancer, chronic obstructive pulmonary disease or chronic heart failure were purposefully recruited at 22 IPC-i in Belgium, Germany, Hungary, the Netherlands and the United Kingdom in the course of the project “Patient-centred palliative care pathways in advanced cancer and chronic disease” (InSup-C).

Semi-structured interviews (*n* = 156) and 87 quantitative questionnaires (CRA, POS, CANHELP Lite) were conducted with family carers. Interviews were analysed with transnationally agreed thematic codes (MAXQDA or NVivo). Statistical tests (SPSS) were carried out in accordance with the characteristic value of the items and distributions.

**Results:**

On average, quantitative data showed moderate burden, but the qualitative findings indicated that this burden might be underrated. There is some evidence that IPC-i with well-developed professional care networks and communication systems relieved family carers’ burden by direct and indirect interventions; e.g. provision of night shift nurses or psychological support. Needs of family carers were similar in all participating countries. However, in all countries IPC-i mostly offered one-off events for family carers, lacking systematic or institutionalised support structures.

**Conclusions:**

Data suggest that, most IPC-i did not pay enough attention to the needs of most family carers, and did not offer proactive care and access to supportive resources to them (e.g. training, respite care, access to resources). We recommend recognizing family carers as part of the ‘unit of care’ and partner in caregiving, to improve their knowledge about, and access to, and the support available.

## Background

Most people with non-curable life-limiting disease want to remain at home as long as possible [1–3]. In some European countries patients and family carers are also often obliged to do so due to cutbacks in residential and professional home care [3]. As a consequence, being cared for at home is highly dependent on the circumstances of an overall home care situation, institutionalised professional support system and availability as well as commitment of family carers^1^ [1, 4, 5]. Van Groenou and de Boer, among others, draw attention to the fact that in European countries, an increase of persons requiring palliative care coincides with a “shrinking family size, the increased labour market participation of women and the rising retirement age”. These societal changes affect the availability of and pressure to informal carers and therewith the care situation of patients with non-curable disease [3, 6, 7].

### Family carers’ overall situation

National and international level information about family carer backgrounds [8–10], their responsibilities, perceived burdens and rewards, unmet needs as well as the effects on carers’ quality of life (QOL) are at the centre of research interest [6, 8, 9, 11, 12]. Providing informal care usually lasts until the death of a patient, which makes the situation for family carers even more demanding [13].

The overall physical and psychosocial strains and deterioration of carers’ QOL are well recognised in the literature [14]. Physical and mental strains include sleep deprivation, tiredness, anxiety and depression [9, 15–18]. Even though different practical and other types of support from specialized multi-professional palliative care teams may be offered to patients and their family carer, the authors of these studies describe a general unpreparedness of family carers to cope with the overwhelming challenges of providing care and sometimes to maintain caregiving until the end [19]. In addition, family carers of older patients are often elderly people themselves [7, 20, 21]. Family carers may be considered as ‘hidden patients’ as Kristanjson and Aoun described them earlier. These authors, furthermore, called attention to their psycho-social and physical burdens [15].

### What is integrated palliative care?

In the European healthcare sector, palliative care was established to meet the needs of terminally ill patients. Across Europe there are different models of palliative care, and types of palliative care services to support family carers looking after patients that wish to die at home [22]. Patients who want to be cared for at home are highly reliant on the quality and continuity of support provided by professionals, volunteers and family carers. The concept of integrated palliative care (IPC) has been introduced as an innovative and progressive way of providing care that is more appropriate to patients’ and family carers’ needs. As there is no unanimously agreed definition of IPC, the InSup-C project collaborators developed a working definition as follows:

“Integrated palliative care involves bringing together administrative, organisational, clinical and service aspects in order to realise continuity of care between all actors involved in the care network of patients receiving palliative care. It aims to achieve quality of life and a well-supported dying process for the patient and the family in collaboration with all the caregivers, paid and unpaid.” [23]

The comprehensive approach of palliative care aims to maintain the best possible well-being of patients and their family carers. For this purpose, IPC goes beyond the scope of standard healthcare where healthcare professionals (HCPs) act as single entities in an uncoordinated and multifaceted care situation. An integrated person-focused palliative care approach for the patient and his/her social environment care “is a ‘complex intervention’ where management and organizational processes to support integrated care occur at many levels^2^ simultaneously” [24].

However, as set out in the protocol article of the InSup-C project, initiatives using an integrated palliative care (IPC-i) approach show promising results in how they reduce fragmentation and enhance continuity of care as well as quality of care. Within the InSup-C project, IPC-i were differentiated from other PC initiatives and defined by being an established local and multidisciplinary PC collaboration with at least two different organizations and their HCPs providing direct patient care. The initiative was either targeting one or more of the following diagnostic groups: advanced cancer, COPD, CHF [23].

Since, within the literature, it is largely unknown how family carers experience integrated palliative care, with this paper we aim to investigate 1) the burdens and rewards associated with family caregiving within IPC networks, and 2) the effects of IPC-i support systems and interventions for family carers.

## Methods

### Design

This study is part of a multiple embedded case study, conducted by the InSup-C project consortium. This study has a mixed-method design [25]. Mixed methods were chosen to increase clarification and complementarity of the results from different methods as well as to extent breadth and scope of the inquiry. Furthermore, we were aiming at convergent validity: this occurs when two different instruments those aim to measure the same phenomenon, show strong coherence [26–28]. That fore the same person was interviewed with an in-depth interview (qualitative part) and standardised questionnaire (quantitative part). Data collection lasted from July 2014 until November 2015 in Belgium (BE), Germany (DE), Hungary (HU), the Netherlands (NL) and the United Kingdom (UK). We used descriptive statistics and determined relationship measures in the quantitative part [23] to establish an overall picture about family carers’ situation. In the next step we utilised our qualitative data material to capture a more holistic understanding of their actual situation [26, 29].

### Study population

In five European countries, IPC-i were selected based on a standardised selection criteria such as locally established networks, collaboration structures and multidisciplinary teams [23]. After the selection of IPC-i, within these IPC-i adult patients with advanced cancer, COPD or CHF and without cognitive and linguistic barriers were identified by their treating physician or other professionals. The patients were asked to identify the person who takes care of and supports them for most of the time. Some of the purposively recruited patients were living alone and a family carer was not available, as a result. General criteria for an interview with an adult family carer were linguistic and cognitive ability to answer the questions.

Furthermore, though family carers are mainly female and kin, such as (spousal) partners and children [7, 30], the use of the term ‘family carer’ in this paper, includes friends and other people who are significantly involved in a patient’s care network [31]. When a family carer showed interest and before starting the interviews, further information was sent.

### Measurement

#### Quantitative study

Family carers were asked to complete questionnaires at the initial contact (baseline) and each month over a period of 3 months (follow-up interviews). In addition to items on demographic and epidemiological data, three standardized questionnaires were used. The Palliative Care Outcome Scale (POS) – version 1 measured patients’ quality of life (QOL) and perceived symptoms from the family carers’ perspectives [32]. The Caregiver Reaction Assessment (CRA) scale helped to determine both burden and reward of family carers’ caregiving with 24 questions on five dimensions: impact on schedule, impact on finances, lack of family support, impact on health and impact on self-esteem [33, 34].^3^ The third questionnaire was the Canadian Health Care Evaluation Project Questionnaire (CANHELP Lite) version for palliative care which aims to measure family carers’ satisfaction with end-of-life care. From the CANHELP Lite questionnaire only the relevant questions about contact between HCPs and family carers [35] were used. The quantitative analyses are based on the baseline questionnaires due to panel mortality.

#### Qualitative interview study

The international InSup-C project team developed an interview guide before starting the interviews, which was piloted by two project members and two family carers per country. In the interview, questions regarding patients’ problems and needs and family carers’ experiences of contacts and collaboration with and between professionals were discussed. To visualize the care network a card technique supported the family carers in describing complex experiences. For the interviewee, the researcher wrote down all persons, who were involved in the care network on cards, which then were used for further sorting and exploration of entities within the care network [36]. Interviews were held twice of which the second and final interview was held 3 months after inclusion date. If a patient died, the bereaved family carers was only interviewed if (s)he estimated that (s)he was able to in face of emotions, e.g. grief. The average duration of an interview was 1 hour. Interviews were audio-recorded and transcribed verbatim. All face-to-face interviews were conducted in the country languages and were mostly held at the informants’ homes.

### Analysis

#### Quantitative data

Missing values: Item-non-response technique was a replacement by mean scores of the other items when a minimum of 40% of items in the CRA subscales or POS summary scores had been completed. Otherwise these mean scores were excluded. CANHELP Lite questionnaire’ subscales were excluded if more than half of the responses for that domain were missing [35].

Using SPSS (Statistical Package for the Social Sciences version 22), in dependence of scale level and distribution different statistical test procedures were applied (Pearson correlations coefficient, T-test, ANOVA-test). The variable ‘relation between patient and family carer’ was re-coded from four to three values.^4^ Family carers’ age was computed by subtracting the variable ‘record creation date’ minus ‘date of birth’.

We investigated correlations between family carers’ burden and demographics, patients’ QOL (with the POS summary score) and family carers’ experiences with care (with the five CANHELP Lite questions). Effect sizes were determined according to guidelines of behavioural sciences; a correlation coefficient of 0.1 was considered small, a correlation of 0.3 as medium or moderate power and an absolute value of 0.5 as large [37]. We rejected a null hypothesis at level α, when the *p*-value was less than or equal to 0.05.

#### Qualitative data

Analysis consisted of two major parts. The first part was a general national analysis in which every country analysed the interview data by performing content analysis in several steps. First, main themes about the organizational structure of and relationship within the PC networks, patients’ and family carers’ needs, problems and solutions were deductively applied to several interview transcripts. These themes were derived from the interview protocol and covered the broad scope of the InSup-C project [24]. Second, researchers identified additional codes from text segments which were then clustered into (sub)codes. During international meetings, codes were discussed and compiled into an agreed code book. Intercoder reliability was aimed by discussing ambiguous text fragments in the international teams [38, 39]. Data saturation was reached when no new codes derived from analysing the data. Although the first step was deductive, the coding process was largely inductive and followed the conventional content analysis [29]. The software NVivo 10 and MAXQDA were used to aid the qualitative analysis.

The second major step was an in-depth analysis of family carer data. The main researchers GA and AE asked the international team to analyse their country-specific data by focusing on family carers’ tasks, burden, rewards, and collaboration with IPC-i by making use of the relevant codes from the codebook and by providing interview quotes. Furthermore, team members were asked for an overall impression of the family carers’ situation and general background on facilities for family carers in the countries. Answers had to be returned in English. GA and AE interpreted, compared and analysed the national data, verified their results with the international team, and circulated a first draft article to assure right interpretation of the data and thus, rigour of the findings. Findings of this study are explorative and descriptive, since the scope and design of the greater InSup-C project were very broad [40]. Illustrative quotes were chosen to exemplify the rich descriptions of our findings.

## Results

### Family carers’ characteristics

For the qualitative part, 92 semi-structured baseline, 50 secondary and 14 bereavement interviews were available (total *n* = 156 interviews). Almost half (46%, *n* = 40) of the family carers stopped prematurely either because they signed out or the patient died. After data cleaning procedures among the 87 respondents 27% (*n* = 23) only finished the baseline questionnaires, 14% finished both baseline and month 1 (*n* = 12), 6% (*n* = 5) participated till month 2 and 54% (*n* = 47) completed the entire period. Family carers’ characteristics and some basic data about the patients they cared for are presented in Table 1.

**Table 1:** Characteristics of family carers and patients

	BE (*n* = 13)	DE (*n* = 10)	UK (n = 13)	HU (*n* = 30)	NL (*n* = 21)	Total (*n* = 87)
*Carer’s age* (mean, SD)	63 (10)	60 (14)	63 (13)	56 (15)	62 (11)	601 (13)
*Gender* (no. female)	6 (46%)	7 (70%)	8 (62%)	24 (80%)	14 (67%)	59 (68%)
*Diagnosis of patient*						
Cancer	10 (80%)	10 (100%)	5 (39%)	11 (37%)	15 (73%)	51 (59%)
CHF	/	/	1 (8%)	7 (23%)	1 (5%)	9 (10%)
COPD	3 (20%)	/	7 (54%)	12 (40%)	5 (23%)	27 (31%)
*Who is patient’s carer?*						
(un)married partner	10 (83%)	4 (40%)	6 (50%)	16 (53%)	15 (71%)	51 (60%)
child	2 (17%)	6 (60%)	2 (17%)	9 (30%)	2 (10%)	21 (25%)
other	/	/	4 (33%)	5 (17%)	4 (19%)	13 (15%)
*Do carer and patient live together?* (no. yes)	10 (83%)	4 (40%)	7 (58%)	22 (76%)	15 (71%)	58 (69%)
*Patient’s age* (mean, SD)	67 (11)	73 (9)	67 (9)	69 (9)	69 (8)	69 (9)

Source: Own data set and own calculations, family carers: *n* = 87

The mean age was 60 years and 68% were female. The majority of family carers were partners or spouses and the majority lived together with the patient. Of the patients cared for, almost 60% suffered from incurable cancer, 10% had CHF and almost one third was diagnosed with COPD.

### Quantitative findings

As Table 2 shows, family carers from our study sample, in general, experienced moderate burden from taking responsibility for the care of a person in need of palliative care. However, the standard deviations show considerable variations. Self-rated positive impact on self-esteem was rated relatively high. Results show prominently that the worse patients’ health (higher POS scores) was associated with more burden on several dimensions of family carers’ CRA. Furthermore, the relation between family carer and patient appeared to be of influence. The health of (un)married family carers (*n* = 50, $\overline{x}$ =2420) was significantly worse than of ‘others’ (*n* = 13, $\overline{x}$ =1731). Finally, it appeared that the older the patient the less rewarding family carers considered taking care of their proxies in form of self-esteem.

**Table 2:** Descriptive statistics of the CRA scale and correlations between CRA and demographic data and POS summary score

		Gender (male, female)		Disease (cancer, COPD, CHF)		Living together (yes, no)		Relation FC-patient (partner, child, other)		Comorbidity patient (yes, no)		Family carers’ age		Patients’ age		POS sum score	
	Mean (SD)^a^	*t*(df)	*p*-value	*F*(df1, df2)	*p*-value	*t*(df)	*p*-value	*F*(df1, df2)	*p*-value	*t*(df)	*p*-value	*r*	*p*-value	*r*	*p*-value	*r*	*p*-value
Disrupted Schedule	2,99(,96)	-1,08 (83)	,29	,19 (2,82)	,82	,82 (80,00)	,42	,89 (2,80)	,42	,08 (65,60)	,94	,07	,51	,06	,58	,33	,00**
Financial problems	2,43(1,00)	-,93 (80)	,36	,82 (2,79)	,44	1,61 (77,00)	,11	1,15 (2,77)	,32	,36 (80,00)	,72	-,07	,54	-,09	,42	,14	,21
Lack of family support	2,06(,91)	-1,20 (82)	,23	,33 (2,81)	,72	1,37 (79,00)	,17	1,06 (2,79)	,35	-,25 (82,00)	,80	-,13	,25	-,01	,97	,24	,03*
Negative impact on health	2,26(,88)	-1,06 (83)	,29	,63 (2,82)	,53	1,82 (80,00)	,07	3,37 (2,80)	,04*	,07 (83,00)	,94	,10	,37	,05	,65	,33	,00**
Self-esteem	4,42(,58)	,26 (82)	,79	,35 (2,81)	,71	,81 (34,47)	,43	,49 (2,79)	,62	-,63 (82,00)	,54	-,13	,26	-,26	,02*	,08	,49

Own data set and own calculations, *n* = 87; ^a^ theoretical range: 1 (strongly disagree) - 5 (strongly agree); * *p* ≤ = 0.05, ** *p* ≤ = 0.01

Table 3 shows that family carers, in general, were very satisfied with the way they were treated and supported by doctors, nurses and other healthcare professionals from IPC-i. Again, individual family carer satisfaction varied as standard deviations indicate. The table also shows how family carers’ burden could be alleviated or prevented by IPC-i. Especially, consistency of information supply about the patient’s condition was related to lower burden on several domains of the CRA. Furthermore, higher satisfaction with the role in decision making was related to the impact of caregiving on health. All correlations, however, were small or moderate.

**Table 3:** Descriptive statistics of family carers’ satisfaction with communication with HCPs (CANHELP Lite) and correlations between CANHELP Lite and CRA

		Disrupted schedule		Financial problems		Lack of family support		Negative impact on health		Self-esteem	
	Mean (SD)^a^	r	*p*-value	r	*p*-value	r	*p*-value	r	*p*-value	r	*p*-value
QB. The way the FC was treated by health care professionals	3,14(,85)	,02	,86	-,16	,16	-,16	,15	-,01	,96	-,13	,26
Q5. The extent to which health care professionals were compassionate and supportive	2,99(1,06)	-,07	,57	-,15	,21	-,21	,06	-,07	,56	-,03	,82
Q16. Consistency of information about patients’ condition	2,93(1,07)	-,24	,04*	-,29	,01*	-,37	,00**	-,26	,02*	-,06	,62
Q17. Listening to FC’s opinions	3,03(1,06)	-,07	,55	-,18	,12	-,24	,04*	-,15	,20	-,00	,97
Q20. Role in decision-making about PA’s medical care	3,07(,91)	-,09	,47	-,04	,72	-,23	,06	-,24	,04*	,15	,22

Own data set and own calculations, *n* = 87; ^a^ theoretical range: 0 (not at all) - 4 (completely); *p*-values: * *p* ≤ = 0.05, ** *p* ≤ = 0.01

Qualitative findings below will provide a more nuanced and elaborate picture of the individual family carers and how they experienced their burden or contact and communication with healthcare professionals.

### Qualitative findings

#### Family carers’ perceived burden

In all IPC initiatives family carers stated a range of current health issues at the time of the qualitative interviews, especially older family carers who listed pain (e.g. knees, back), cardiac dysrhythmia, insomnia, shortness of breath, restlessness and feelings of guilt and anxiety as forms of physical and mental distress.

*“Even of late my health’s suffering a little bit […] I’m getting very tired and getting dizzy spells and… and I know it’s just stress and I am trying to do everything (…).” (FC in UK, female, home care and hospice day care).*

*“I have cardiac arrhythmias and partially shortness of breath; I am not the healthiest one and I am truly trying to stay afloat (…).” (FC in DE, male, home care).*

*“I have pain in my back, always pain in my legs. I have had shingles and I am taking pills against anxiety, to calm me down.” (FC in BE, female, home care).*

As a consequence of concurrent responsibilities, control over their own health and needs was often suppressed to provide patients with the best possible quality of care at home. Some of the older family carers questioned whether their own health conditions would allow them to continue performing caregiving activities. The overall tenor was that a further worsening of own health would lead to decreased caregiving ability at home.

*“I can‘t lift her anymore. In the morning, the children are gone, so that I am already in the morning in troubles. (…) just as an example I can‘t do this anymore.” (FC in DE, male, home care).*

Looking at family carers’ own conditions, one family carer reflected on how the ongoing process with the patient explicitly affected her personal dietary needs, which resulted in a distinct loss of weight:

*“I was in a different world really. I’d been caring for him for so long that I think my… my own self had kind of gone into the background … I’ve lost a lot of weight because I suppose I haven’t been looking after myself at all. I mean I’m not aware that I’m not looking after myself but obviously it’s taken its toll, which is bound to happen.” (FC in the UK, female, home care).*

In the course of the interviews, topics of psychological burden and social isolation caused by caregiving responsibilities were mentioned very often. Most of the family carers yearned for additional free time or respite care.

*I: What are your needs?*

*C: Rest, mentally and physically. I am never at ease, not when I am with him and not when I am without him.*

*I: Do you ever go out?*

*C: Never (FC in BE, female, home care).*

### Rewards and motives

Qualitative findings did not extensively describe rewards. A family carer stated that he learned a lot about himself in the process. Other quotes were about experiences of connectedness between patient and family carer. Family carers related this to special moments, when they felt gratitude or happiness that the person cared for was still alive.

*“I do not think caring for my husband is very hard, well, it is difficult to see him deteriorate, but I don’t mind caring for him. Lots of people tell me: ‘Remain strong!’ and yes, the fact that I can do a lot for him keeps me strong. Probably I will get problems when he is not here anymore to care for.” (FC in BE, female, home care).*

In bereavement interviews some family carers spoke modestly or even with humility of what they have achieved:

*“That was so important for me. He died in the home where he was born.” (FC in DE, female, home care).*

Furthermore, throughout the interviews a number of motives for caregiving became evident. It seemed that family carers tended to not focus on their own interests and well-being, finding ‘the other’ more important and considering informal care a ‘duty’. In some cases this was a result of reciprocal action, meaning that the family carer received help from the patient before they fell ill. In other cases, caring was considered as only natural. In a few cases caregiving was seen as a heavy burden which was not always taken on willingly, particularly when there were no other solutions for the provision of care to be seen. Most of the time family carers – for diverse reasons – tried to cope and care on their own as long as possible. This was especially mentioned by family carers living with the patient, most of whom were spouses. There was also the motive of love, which in some cases buffered or reprioritized the negative aspects of caring.

*“I am helping as much as possible with everything she needs. And when she asks me, I say to her “honestly, I do so with love”. I wouldn’t want it any other way, although it is tough.” (FC in the NL, female, home care).*

#### Access to IPC services, personnel and information

In the qualitative results family carers who were frequently in contact with an IPC-i team stated high satisfaction with assistance. Many quotes indicated that an overall gratitude towards IPC-i is due to a pro-active support for patients. In conjunction with this we received a lot of positive comments in all countries. Thus, IPC-i involved in this study gained a lot of trust: those who have a contact person for every upcoming situation 24/7 gave them a feeling of confidence in dealing with the home care situation.

*“Those nurses make me feel at ease. (...) in the beginning you have to get used to the different faces, but now we have the same ones during the week and it changes a little at the weekends. They are five in total, no, they don’t bring in new staff, so we got used to them all.” (FC in BE, female, home care).*

*“We have a good relationship with the doctors. My mom always got answers to her questions. If she needed any treatment, her doctor informed her about the treatment and the possible side effects. If I perceive anything unusual, I can ask the doctor.” (FC in HU, male, home care).*

However, qualitative findings showed that there were some flaws in access to information, personnel and services in all countries. These concerned: preparedness for transitions between care settings, continuity of personnel, timely and pro-active information and mobilisation of services, lack of access to specific care if this was not covered for the specific disease (non-cancer mostly) and responsiveness of HCPs.

Within our data most of the IPC-i showed limitations in recognising family carers’ needs and did not seem to provide pro-active support for family carers (education and training, respite care, access to resources etc.).

*“There are a lot of things that can be arranged for medical care, but you have to arrange everything yourself. They tell you or they don’t, and you always have to do it yourself.” (FC in BE, male, home care).*

Getting appropriate information about care in the last days of life can help to tackle care conditions that had caused feelings of uncertainty and helplessness at this sensitive time. A female respondent explained how receiving information about diet and nutrition in palliative care from an IPC physician was seen as an essential component to reduce the carer’s stress and lack of self-confidence.

*“And then he (IPC physician) gave me this brochure about the last weeks and days. (...) And that was very helpful and I have given it to our visitors to read it. (...; The) doctor said to my husband, you don’t have to eat. If you like to eat, it’s fine, but you don’t have to eat. Usually, you don’t hear that. You always hear how people repeatedly say you still have to eat (...).” (FC in DE, female, home care).*

Furthermore, most family carers within an IPC network expressed their need for information to overcome their uncertainties after discharge from hospital or in the last days in life:

*“I was like, I would have appreciated if they told earlier. Then we could have adapted our reactions to him. […] He just knew. If we would have known earlier, we could have handled the situation differently.” (Bereaved FC in the NL, female).*

#### Practical and psychosocial support

Qualitative findings showed that HCPs from IPC-i provided practical and some emotional support to reduce family carers’ responsibilities. Practical and medical interventions for the care of a patient were offered most frequently: cleaning, medical home care or instructions about easy nursing tasks by either the initiative or the general practitioner. Emotional support from HCPs, however, appeared to be more ambiguous. Many family carers reported that HCPs were mostly supportive in their approach: being attentive and genuine, taking time and creating a trusting environment. Feeling involved and listened to in matters of care and decision-making was considered helpful by family carers, but was not an experience shared by all. During the interviews, offers of specialised psychological or spiritual help for family carers were reported only rarely.

Respite care as a form of support was only offered in a few cases. Especially one case, however, showed that it released family carers from stressors and strains at a personal level:

*“We did have Hospice coming… when he was seriously ill, we had Hospice coming in at nights for two nights a week and then, as he got better, there wasn’t a need. And they came out and they assessed him and they said we’ll still send someone on a Friday so that you can shop which, you know, is good.” (FC in the UK, female, home care).*

Family carers who participated in this study mentioned the usefulness of practical and psychological support provided by IPC services. An example showed how an acknowledgment of the family carer’s role in the care network improved the carer’s overall situation:

*“(I): And then you said they were doing something for you personally now, what’s that? (FC): Yes. It’s complementary therapy, it’s like aromatherapy, massage. (…) It’s lovely, yeah, it’s really nice.” (FC in the UK, female, home care).*

Active offers to support the well-being of family carers were provided ad hoc rather than as systematic interventions following ‘care pathways for family carers’ including needs assessment on a regular basis developed or used by IPC-i.

## Discussion

The aim of this paper was to analyse the family carers’ situation within an IPC network in five European countries (BE, DE, UK, HU and the NL). Our quantitative data showed moderate burden with a high positive impact on family carers’ self-esteem, but the qualitative data underlined a higher risk of physical and psychological health issues as well as an actual deterioration of health, especially in older or spousal carers. This higher risk on (over)burden among spousal or older carers in general and when patients’ QOL decreases is consistent with other results in this field [17, 41, 42].

The differing quantitative and qualitative findings on family carers’ burden can be interpreted as a consequence of change in family carers’ perceptions. They might have focused on the patient rather than on changes within oneself and might have compared their own health issues with that of the patient. Furthermore the motive of love for caring, might have buffered or reprioritized negative aspects of caring, as has been highlighted in other studies [43–45].

In the literature several motives for caring are seen as potential risks for burdening, e.g. personal bond between patient and family carer [42], desire to prevent residential care, family carers wish to take on most of the care themselves and to keep on doing this as long as possible [46] or centering care around the patient by considering informal care a ‘duty’ [8, 47, 48]. These intrinsic motivations include the risk of gradually adapting to and growing with circumstances without realising overburdening is lurking [48]. By asking about their motives and availability of external support, HCPs could start a conversation about desires within and risks of caregiving.

From this study, it appeared that HCPs from both IPC-i and from outside IPC-i provided some practical and emotional support to family carers to release their responsibilities. Our quantitative and qualitative findings confirmed that reliable and consistent transmission of information and regular contacts with IPC-i were important to lighten feelings of uncertainty. The same conditions more or less accounted for access to services and personnel. Literature also made notice of these conditions, but most often as things that are lacking in practice [49–53]. Within our study, family carers involved in IPC-i appeared quite satisfied with information transfer and other communication with HCPs.

As was shown in our results and in literature support at home is reported to be the most common intervention to support family carers [54, 55]. Qualitative results, however, tend towards the importance of respite care, though options to delegate responsibility or get a break were mentioned only rarely. Improvements in IPC-i could focus more on respite care as this has been proved effective in reducing family carers’ burden [53–57].

Even though IPC-i aim at taking care of both patients and family carers, the barriers to offer pro-active care for family carers seem to lie within the healthcare systems. The allocation of help still seems to depend mostly on whether or not family carers clearly express their need for support. Active interventions on an operative level by IPC-i, such as the two well illustrated examples in this study, are singular events even within an advanced healthcare system. In view of our results, HCPs of ICP-i need to recognise the gradual reduction of carers’ welfare in a timelier manner and consider patients and family carer as part of the ‘unit of care’. Ongoing pro-active carer support including respite care, individualised training courses, counselling services and assessing their healthcare condition on regular basis may help to strengthen and empower family carers for their caregiving duties in advance [58]. HCPs clarifying and explicitly offering support, furthermore, would increase both family and patient satisfaction [59]. Also, they would like to be identified and recognized in their role by HCPs and community, too [53, 60]. In light of this, it is interesting that local or community resources for support were not often mention by the respondents in our study.

### Strengths and weaknesses

The study’s sample size as well as its international character increases the transferability of the findings. However, our study also has some limitations. Since the scope of the research project InSup-C was very broad, this study was mostly explorative and hypothesis-generating. Findings of this study as well as suggestions for clinical practice need to be tested and investigated by further research. Since we used selected IPC-i for the gathering of data, findings cannot be generalized for all countries. Another limitation is that quantitative statements about satisfaction with care were based on individual questions of the CANHELP Lite. To resolve this, these statistical results were complemented by qualitative findings. Finally, it might be that, during interviewing, researchers unintentionally focused on burden of care. This would explain the discrepancies between qualitative and quantitative results, but would indicate a data bias. Focusing on the negative side of caregiving is a trend within literature as well [42, 61].

## Conclusions

Needs of family carers were similar in all participating countries who on average experienced a moderate burden from caregiving, which seems however underrated in the light of the findings of the qualitative analysis. Consistent communication towards family carers, in particular information sharing, came afore as being inversely related to carers’ burden, stressing the importance of communication in end of life care. Qualitative findings indicate a need for proactive care for family carers, for example provision of respite care, training, and access to resources. This study recommends recognising family carers as part of the ‘unit of care’ and partners in caregiving at the same time, to improve their access to support and their knowledge about available support options.

## Abbreviations

BE: Belgium
CANHELP Lite: Canadian Health Care Evaluation Project
CHF: Chronic Heart Failure
COPD: Chronic Obstructive Pulmonary Disease
CRA: Caregiver Reaction Assessment
DE: Germany
FC: Family Carer(s)
HCPs: Healthcare professionals
HU: Hungary
IPC: Integrated Palliative Care
IPC-i: Integrated Palliative Care initiative(s)
NE: the Netherlands
PC: Palliative Care
POS: Palliative Care Outcome Scale
QOL: Quality of Life
SD: Standard deviation
UK: United Kingdom

## Footnotes

1 In line with the National Institute for Health and Clinical Excellence (NICE) a person with a kinship or marriage as well as a person with a close relationship (socially and emotionally such as neighbours and friends) are specified as family carers in this paper

2 Goodwin refers to the concept of Valentijn et al., where levels of an integrated person-focused palliative care approach should consider integration processes at “the macro- (system integration), meso- (organisational, professional) and micro-level (clinical, service and personal integration)”.

3 We determined a good internal consistency for the POS summary score (Cronbach α = 0.709), CRA subscales (α between 0.741 and 0.836)iii, and CANHELP Lite’s subscales (α from 0.830 to 0.918) and its overall mean score (α =0.933). To increase the internal consistency for the index ‘impact on self-esteem’ within our sample one of the items (nr. 21) had to be removed

4 The variable ‘relation between patient and family carer’ was re-coded from four variable values (1 ‘(un)married partner’, 2 ‘child’, 3 ‘parent’ and 4 ‘other’) to three values (1 ‘(un)married partner’, 2 ‘child’, 3 ‘other’), due to one interviewed ‘parent’ among all countries.
